# Mediterranean Spotted Fever: Current Knowledge and Recent Advances

**DOI:** 10.3390/tropicalmed6040172

**Published:** 2021-09-24

**Authors:** Nikolaos Spernovasilis, Ioulia Markaki, Michail Papadakis, Nikolaos Mazonakis, Despo Ierodiakonou

**Affiliations:** 1 School of Medicine, University of Crete, 71003 Heraklion, Greece; nikspe@hotmail.com (N.S.); papadakis.mixal@gmail.com (M.P.); nikosmazwnakis13@hotmail.gr (N.M.); 2 “Trifyllio” General Hospital of Kythira, 80200 Kythira, Greece; tzouliamar95@gmail.com; 3 Department of Social Medicine, School of Medicine, University of Crete, 71003 Heraklion, Greece; 4 Department of Primary Care and Population Health, University of Nicosia Medical School, Nicosia 2417, Cyprus

**Keywords:** *Rickettsia conorii*, Rickettsiales, Mediterranean spotted fever, tick-borne disease, *Rhipicephalus sanguineus*

## Abstract

Mediterranean spotted fever (MSF) is an emerging tick-borne rickettsiosis of the spotted fever group (SFG), endemic in the Mediterranean basin. By virtue of technological innovations in molecular genetics, it has been determined that the causative agent of MSF is *Rickettsia conorii* subspecies *conorii*. The arthropod vector of this bacterium is the brown dog tick *Rhipicephalus sanguineus*. The true nature of the reservoir of *R. conorii conorii* has not been completely deciphered yet, although many authors theorize that the canine population, other mammals, and the ticks themselves could potentially contribute as reservoirs. Typical symptoms of MSF include fever, maculopapular rash, and a characteristic eschar (“tache noire”). Atypical clinical features and severe multi-organ complications may also be present. All of these manifestations arise from the disseminated infection of the endothelium by *R. conorii conorii*. Several methods exist for the diagnosis of MSF. Serological tests are widely used and molecular techniques have become increasingly available. Doxycycline remains the treatment of choice, while preventive measures are focused on modification of human behavior and vector control strategies. The purpose of this review is to summarize the current knowledge on the epidemiology, pathogenesis, clinical features, diagnosis, and treatment of MSF.

## 1. Introduction

*Rickettsia conorii* is a vector-borne, obligate intracellular bacterium causing Mediterranean spotted fever (MSF), mainly in the Mediterranean area and the surrounding countries [1]. This clinical syndrome is characterized by diverse clinical manifestations, presenting with both typical and atypical features, making the early diagnosis of the infection rather challenging, thus requiring extra suspicion in the triage of the febrile patient presenting within or from endemic areas. The purpose of this review is to summarize the current knowledge of this zoonotic vector-borne infection and to present future prospects and progress made in the rickettsial field.

## 2. Epidemiology

MSF was firstly reported in Tunisia in 1910 as a clinical syndrome named “fièvre boutonneuse” or macular fever, due to the accompanying rash [2]. Shortly after, cases of MSF were detected in other Mediterranean countries while the first description of the inoculation eschar known as “tache noire” was made in 1927 [1]. Nowadays, MSF is considered endemic in the Mediterranean basin but it can also afflict returning travelers from this area [3,4].

*R. conorii* has been identified as the responsible agent of MSF and was firstly described by Brumpt in 1932 [5]. *R. conorii* is part of the *Rickettsiaceae* family, member of the order Rickettsiales, within the α-proteobacteria class. Such bacteria bear a set of common traits, as they are Gram-negative, strict intracellular, short rods that retain basic fuchsin when stained by the method Gimenez [6].

Recent phylogenetic studies, based on whole genome sequencing, have demonstrated a more detailed classification for these bacteria. According to these studies, the species of the genus *Rickettsia* can be classified into four groups: a spotted fever group (SFG) (*R. rickettsii*, *R. conorii* and others); a typhus group (TG) (*R. prowazekii* and *R. typhi*); an ancestral group (*R. bellii* and *R. canadensis*) and the recently formed transitional group (*R. akari*, *R. australis* and *R. felis*) [7,8]. Moreover, further technological advances in molecular genetics and the introduction of genomics and transcriptomics have reshaped the rickettsial field and allowed for further and more detailed approaches regarding rickettsial taxonomy [9,10]. Zhu et al. demonstrated that the genotypic variability among *R. conorii* strains, which exhibited different epidemiological and clinical manifestations as pathogens, allowed further division of the *R. conorii* species and the creation of the following subspecies within the *R. conorii* complex: *R. conorii caspia*, *R. conorii israelensis*, *R. conorii indica,* and *R. conorii conorii* [11]. *R. conorii conorii* is the causative agent of MSF [11].

A common trait of the *Rickettsia* species of the SFG is that they are mainly transmitted to vertebrate hosts by arthropod vectors, mostly ticks [2]. Ticks are hematophagous arthropods that can potentially parasitize all vertebrates and are categorized in two major families, the Ixodidae (hard ticks) and the Argasidae (soft ticks), with different morphological features and feeding habits. Ixodid ticks are the main SFG rickettsiae vectors [5]. The life cycle of hard ticks consists of developmental stages and the transition through those stages requires the tick to attach to a host and feed for several days. Once satiated, the tick detaches from its host and seeks for a resting place to digest its blood meal [5]. After the initial infection, rickettsiae have the ability to multiply in most of the organs and fluids of the tick. The presence of the bacteria in the ticks’ salivary glands is rather important as it enables the rickettsial transmission through feeding [12].

**Figure 1:**
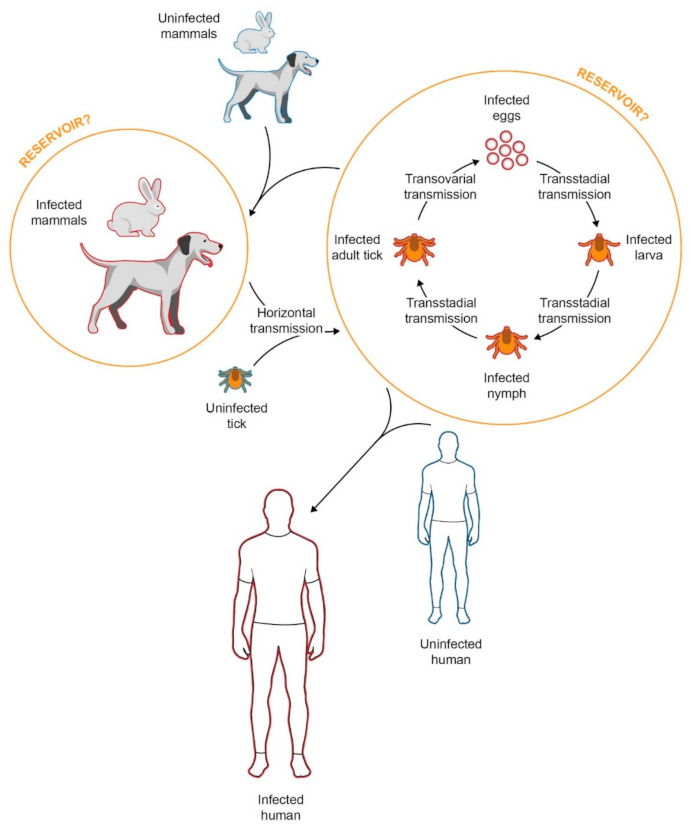
*Rhipicephalus* *sanguineus* can be infected with *Rickettsia conorii conorii* through three main routes: when ticks feed on infected mammals (horizontal transmission), transovarially (vertical transmission), and transstadially (vertical transmission). The transovarial and transstadial transmission of *R. conorii conorii* within the tick population could suggest the potential role of the tick as a reservoir. The true nature of the reservoir of *R. conorii conorii* is not yet fully comprehended, while many authors theorize that the canine population, as well as other mammals, could potentially contribute as reservoirs. The transmission of *R. conorii conorii* to humans is achieved through infected ticks, which are transferred to the human habitat via the canine population.

Currently, three major ways of tick infection with SFG rickettsiae have been described. Ticks may acquire bacteria by parasitizing on infected mammals, a process known as horizontal transmission; bacteria can be also transmitted vertically through transovarial transmission (adult female tick to egg) and via transstadial transmission (egg to larva to nymph to adult tick), thus circulating in all developmental stages; (Figure 1) [5,13]. Direct bacteria transmission from infected to uninfected ticks through co-feeding sites has also been reported, but such events are rather rare [5]. In the MSF setting, it is well established that the arthropod vector of *R. conorii* is the brown dog tick *Rhipicephalus* *sanguineus*, as was initially proposed by Durand and Conseil in 1930 [1]. The tick itself is considered to have a more dominant role in the *Rickettsia conorii* lifecycle, serving as a potential reservoir of the bacteria apart from solely being the vector [14].

The conviction that *Rh. sanguineus* could be a potential rickettsial reservoir has been widely endorsed since the bacteria could be transmitted both transovarially and transstadially in the tick population, thus maintaining a constant cycle [5]. The support of such an argument was exhibited by Blanc and Caminopetros in 1932, who proved the existence of the transovarial route of transmission and suggested that ticks could indeed act as a reservoir of *R. conorii* [1]. Much later, Socolovschi et al. studied the transmission of *R. conorii conorii* over several generations of *Rh. sanguineus*, highlighting that after ten generations from the initial infection of a female tick, there existed 100% transovarial and transstadial transmission in laboratory conditions [15]. However, such high infestations are not observed in nature, as the prevalence of ticks infected by *R. conorii* in the wild is reported to be low. It is estimated that infection rates are as low as <15% and in some studies lower than 1% [16,17,18]. A potential explanation for such observations could be that *Rickettsia* infected dormant ticks may not survive in low temperatures [15]. Complementary, it has been proposed that ticks carrying rickettsiae belonging to the SFG present with reduced fertility and lifespan, while it is also possible that vertebrates may have a stronger impact in the SFG *Rickettsia* species ecology than previously believed, acting as important reservoirs [14,15].

The role of mammals in the prevalence of MSF in endemic areas has been supported by the seroprevalence of *Rickettsia* species in the canine population in certain foci of endemicity [19,20]. Moreover, observations and experimentations that took place early in the history of the disease, in 1930, found that infection of six-week-old puppies with *Rickettsia* infected ticks did not cause any symptoms to the dogs, while their blood could cause typical MSF symptoms when inoculated in humans [14]. In addition to that, similar results were obtained in 1972, when intermittent rickettsemia was detected in dogs after they were infected with a Zimbabwean strain of *R. conorii* without presenting with typical MSF symptomatology [21]. However, such transient rickettsemia makes the canine population a temporary reservoir, potentially unable to contribute to the *R. conorii conorii* cycle efficiently, and probably limiting their role to bringing infected ticks closer to humans [1]. Other mammals that were also found to correlate with *R. conorii conorii* were hedgehogs and small rodents, while the most interesting of those was the European rabbit *Oryctolagus cuniculus* and its impact on the incidence of MSF [1]. The latter relationship was exhibited when a sudden decrease in MSF incidence was reported in France in 1952 which occurred shortly after a decrease in regional rabbit population due to myxomatosis [22]. Additionally, an increase in MSF cases was later observed with the reemergence of rabbit population in 1967 [14]. Despite the aforementioned observations, experimentations and theories, the relationship between *R. conorii conorii* and its vector *Rh. sanguineus* is not yet completely comprehended, while the “rickettsial reservoir” is a controversial subject in current literature and has not yet been definitively deciphered.

The incidence of MSF is not constant. Data from endemic areas of the disease demonstrate that the frequency of MSF cases varies, presenting with peaks and valleys during the past few decades [1,23]. These fluctuations in the incidence of MSF have not yet been attributed to a specific cause. Multiple reasons have been proposed to explain this phenomenon, such as climatic variations from year to year or extreme climatic events, and the use in many studies of non-specific diagnostic methods that could not differentiate *R. conorii conorii* infections from infections caused by other SFG rickettsiae or other subspecies of the *R. conorii* complex which could manifest as MSF-like clinical syndromes [1,23,24,25,26]. However, some Mediterranean countries have experienced a constant increase in the incidence of MSF over the last years [27].

Epidemiological data also reveal that the incidence of MSF seems to follow a seasonal endemicity, as most reported cases in endemic areas seem to emerge in the summer [28,29,30]. High temperatures affect the incidence of the disease by modulating tick behavior. Multiple epidemiological and clinical evidence exists to support this claim. Examples include the emergence of MSF cases presenting with multiple eschars in warmer months and the overall increase in MSF incidence in peaked temperatures [28,31]. This claim was also supported through an experimental model in 2008 which demonstrated that warmer temperatures affect *Rh. sanguineus* habits, aggravating all of its developmental stages to seek hosts more intensively, leading to increased human affinity [31]. Of note, the immature stages of the ticks are significantly smaller than adult ticks and cannot be easily distinguished when attached to human skin [28,29,32].

Another intriguing observation is that *R. conorii conorii* can only be traced within limited regions, whereas its vector *Rh. sanguineus* can be spotted all around the world [1]. Notably, even in endemic areas there seem to exist smaller foci in which, for yet unknown reasons, the disease thrives, while in neighboring regions it does not seem to emerge [1]. In an attempt to explain the prevalence of *R. conorii conorii*, as well as other pathogenic rickettsiae, theories have been proposed trying to elucidate the evolution and the dominance of these bacteria in certain tick populations and therefore geographic regions. The theory of coevolution, for instance, aims to shed light on the origins of tickborne zoonoses by suggesting that the geographic localization of tickborne diseases in specific foci occurs when these areas contain the optimal conditions for the animals involved in the bacteria life cycle to evolve and thrive [5]. Therefore, vectors and hosts of the bacteria are being put through selective pressure so as to coevolve. This theory is endorsed by the dominance of different *Rickettsia* species in different geographic regions all over the world [5]. Furthermore, another evolutionary scenario that has been proposed supports that when tick ovaries get infected with an SFG *Rickettsia*, the molecular-expression profile of the oocytes changes in a way that it blocks a potential second infection from another SFG *Rickettsia* [33]. This phenomenon, described as “rickettsial interference”, could affect the frequency and distribution of certain pathogenic rickettsiae in specific geographic regions [33,34].

## 3. Pathogenesis

**Figure 2:**
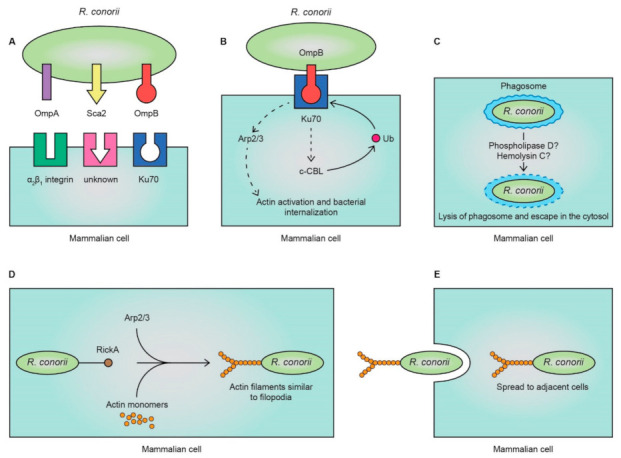
(**A**) *Rickettsia conorii* mediates cellular entry by coordinated interactions between outer-membrane protein A (OmpA) with α_2_β_1_ integrin, surface cell antigen 2 (Sca2) with an unknown receptor, and outer-membrane protein B (OmpB) with Ku70; (**B**) binding of OmpB to Ku70 triggers a host-signaling cascade that involves the activation of c-Cbl and the ubiquitination of Ku70. The distal arm of this pathway activates the actin related 2/3 (Arp2/3) complex which results in actin activation and bacterial internalization; (**C**) the bacteria use membranolytic enzymes, possibly phospholipase D and hemolysin C, to achieve phagosomal escape and gain access to host cytosol; (**D**) bacterial RickA activates the Arp2/3 complex which leads to host cell actin polymerization and the formation of a network of long actin filaments; (**E**) the bacteria can then move to the extracellular space and the adjacent endothelial cells.

Our knowledge concerning the pathogenesis of rickettsial diseases has advanced significantly, yet many questions remain unanswered due to the strict intracellular lifestyle of the bacteria. Several surface cell antigen (*Sca*) genes, encoding proteins similar to autotransporter proteins, have been identified and revealed to play a determining role in rickettsial adhesion to host cells (Figure 2A) [8,35,36]. The *Sca5* gene encodes rickettsial outer-membrane protein B (OmpB), which has been shown to mediate bacterial adhesion to non-phagocytic mammalian cells [37,38]. OmpB is present among species of TG and SFG rickettsiae, including the *R. conorii* complex [39]. *R. conorii* OmpB binds specifically to Ku70 [37]. Ku70 is a component of the DNA-dependent protein kinase complex and was originally thought to be a nuclear protein [40]. However, it has been suggested that Ku70 is present within lipid microdomains on the surface of human target cells [37,41]. Binding of *R. conorii* OmpB to Ku70 stimulates the ubiquitination of Ku70 by recruitment of the E3 ubiquitin ligase c-CBL to the sites of entry (Figure 2B) [42]. The ubiquitination of Ku70 is hypothesized to lead to a cascade of events that ultimately induces host cell actin polymerization that aids in rickettsial internalization [43]. Apart from facilitating bacterial entry, the OmpB has also been associated with evasion of complement-mediated clearance [44].

Another protein that mediates *R. conorii* invasion is outer-membrane protein A (OmpA) by interacting with α2β1 integrin on endothelial cells (Figure 2A) [45]. In addition, it has recently been demonstrated that OmpA serves as a ligand for the fibroblast growth factor receptor 1 (FGFR1) to promote rickettsial internalization [46]. Furthermore, the Sca2 autotransporter protein was also proven to play a critical role in bacterial invasion, a fact that was supported by its presence in the genome of a variety of distinct SFG rickettsiae (Figure 2A) [36]. Arguably, plenty of unidentified interactions between rickettsial surface proteins and host cell surface entities facilitate the bacterial invasion.

Following the internalization of rickettsiae into host cells, the bacteria use membranolytic enzymes, possibly phospholipase D and hemolysin C, to achieve phagosomal escape and gain access to host cytosol (Figure 2C) [47,48,49]. After that, *R. conorii* utilizes the host cell actin cytoskeleton to move within and from cell-to-cell. That is accomplished by a bacterial surface protein called RickA that activates the actin related protein 2/3 (Arp2/3) complex (Figure 2D) [50]. Activation of the Arp2/3 complex induces actin polymerization by creating a nucleation core that results in the formation of a network of long actin filaments that are similar to those present in filopodia [13,50,51,52]. The ability of *R. conorii* to move intercellularly using this mechanism could be a possible immune-evasion strategy (Figure 2E).

The major target cells of *R. conorii* are mainly the endothelial cells lining small and medium-sized blood vessels, but also the macrophages and the hepatocytes [53]. Infection of human endothelial cells with *R. conorii* results in increased vascular permeability, generalized vascular inflammation and edema, and recruitment and infiltration of immune cells by a series of events that are not yet fully clarified [54]. Several vasoactive mediators are produced by endothelial cells during a rickettsial infection [55]. In particular, transcriptional activation of cyclooxygenase-2 (COX-2), that leads to a robust secretion of prostaglandins, takes place [55]. Endothelial cell injury in the context of rickettsioses is thought to be the result of oxidant-mediated cell injury, which was advocated by findings using electron microscopy in addition to enhanced severity of disease in patients with glucose-6-phosphate dehydrogenase (G6PD) deficiency [56]. Moreover, during a phenomenon termed “endothelial activation”, two major signaling cascades, nuclear factor-κΒ (NF-κΒ) and mitogen-activated protein kinase (MAPK), are activated in order to produce proinflammatory cytokines [57,58,59]. These cytokines enhance the expression of cellular adhesion molecules that permit leukocyte recruitment at the sites of inflammation [60,61].

A key component in the host’s response to *R. conorii* is the production of interferon beta (IFN-β) by the infected endothelial cells [62]. IFN-β leads to the activation of the signal transducer and activator of transcription (STAT) family of proteins, which subsequently interfere with rickettsial replication in host cells [63]. This was supported by the enhanced bacterial replication following the induction of IFN-β neutralizing antibodies, while addition of exogenous IFN-β had the opposite effect [62]. Three mechanisms are mainly involved in the killing of intracellular rickettsiae: nitric oxide synthesis; hydrogen peroxide production; and tryptophan degradation [64]. Macrophages, T-lymphocytes and natural killer (NK) cells produce interferon gamma (IFN-γ) and tumor necrosis factor alpha (TNF-α) that act synergistically to induce the production of nitric oxide in endothelial cells [65]. In human macrophages, eradication of the bacteria is accomplished by the production of the enzyme indoleamine-pyrrole 2, 3-dioxygenase (IDO) which degrades and, therefore, limits the availability of tryptophan, resulting in the starvation of the bacteria [64,66]. Dendritic cells (DCs) also appear to play a critical role in the immune response against rickettsial infections. Transfer of *R. conorii*-stimulated DCs to mice led to the rapid increase of CD4+, CD8+, NK cells and IFN-γ production and protected them from lethal rickettsial challenge [67].

However, *R. conorii* has developed sophisticated strategies to evade host immune defenses. As mentioned above, *R. conorii* can infect macrophages, reprogramming their gene expression profile so as to promote its intracellular survival. In further detail, *R. conorii* shifts macrophages towards an anti-inflammatory M2 phenotype, which is primarily accomplished through the modulation of key metabolic pathways [68,69]. At the same time, *R. conorii* induces the expression of several pro-survival genes in macrophages, in order to maintain its replicative niche [70]. In addition, infection of endothelial cells with *R. conorii* in vitro has been associated with the activation of the mechanistic target of rapamycin (mTOR) signaling. It is hypothesized that rickettsiae may exploit this mechanism to evade xenophagy, a form of selective autophagy, that constitutes an important host defense strategy against intracellular bacteria [71].

Nowadays, research on transcriptomics of *R. conorii* aims to shed light on the potential regulatory role of noncoding RNA during the course of rickettsial infections [66,72]. Furthermore, proteomic studies have identified a new protein molecule implicated in various host-rickettsial interactions, that could have a potential diagnostic, as well as prognostic, value [73,74]. Nevertheless, many aspects of *R. conorii* infection pathogenesis remain obscure.

## 4. Clinical Features

The clinical features of MSF reported in the literature vary substantially regarding frequency, duration, and severity. This is probably attributed to the fact that the existence of different subspecies within the *R. conorii* complex and the identification of *R. conorii conorii* as the etiological agent of MSF were only recently described [11]. Because of that, many cases reported as MSF based on non-specific serological assays in previous patients’ series may represent cases of similar but different syndromes caused by other members of the *R. conorii* complex or the SFG rickettsiae.

MSF is characterized by the following classical triad of symptoms: fever, maculopapular rash, and an inoculation eschar at the site of the tick bite [32]. Fever is present in nearly all patients after an incubation period of approximately six days, although it can vary from one to sixteen days [32,75]. Other symptoms that appear early in the course of the disease include headache, arthralgias and myalgias, local lymphadenopathy, hepatomegaly, splenomegaly, and gastrointestinal symptoms [76,77,78].

The vast majority of patients develops a sparse macular rash which later becomes maculopapular and generalized, usually involving the palms and soles and sparing the face [78,79,80]. The rash appears two to three days after the onset of fever but can be delayed until the fifth day. In rare cases (1–4%), it is absent [32]. In some studies, it is described to be petechial in approximately 10% of patients and infrequently it has a form of a vesicular exanthema [77,79].

The characteristic eschar (‘‘tache noire’’) has a variable frequency. In most series it is one of the main clinical manifestations of MSF, presenting in ≥60% of cases [76,78,79], whereas in one retrospective study in Sicilian children from 1987 to 2010 it was present in only 29% of patients [81]. In adults it is usually located on the trunk and the lower and upper limbs, whereas in children it is usually found on the head, neck, and at the auricular region [76,82,83,84]. Cases of multiple eschars are rare, linked to increased aggressiveness of *Rh. sanguineus*, but may also be related to other rickettsioses [30,32,76].

Generally, MSF in children has similar clinical features as in adult patients. However, apart from the differences in the location of the eschar, studies which examine the disease during childhood demonstrate a higher frequency of gastrointestinal symptoms, lymphadenopathy (mainly cervical) and hepatosplenomegaly, and also a lower frequency of headache, arthralgia, and myalgia in children compared with adults [78,82,83,84].

In the majority of cases, MSF presents as a self-limited disease, which persists for 12 to 20 days [32]. However, hospitalization is not uncommon [85]. When treatment is administered, symptoms start to resolve after 48 h and usually complete recovery takes place within 10 days. There is no chronic form of the disease [32]. Regarding asymptomatic infections with *R. conorii*, it is estimated that they have a marked frequency in endemic areas. Seroepidemiological surveys conducted on healthy participants in western Sicily and on blood donors in the south of Corsica revealed a significant prevalence of antibodies against *R. conorii* [86,87].

Despite the fact that MSF usually has a mild course without further consequences, complications have been reported in 1% to 20% of patients and the case fatality rate is 0% to 3% in most published series [88]. Severe cases seem to have a different distribution in time and space and differences in morbidity and mortality rates in the last decades may be explained by the convenience in recognition of severe cases over the years or alterations in strain virulence, but these are only hypotheses [32].

Advanced age, chronic alcoholism, cardiac and respiratory impairment [89], tobacco use [90], diabetes mellitus [23], G6PD deficiency [91], immunosuppression, and delayed initiation of treatment [30] have all been associated with severe MSF. Purpuric rash and abnormal laboratory values such as thrombocytopenia, hyponatremia, hypocalcemia and elevated transaminases have also been correlated with severe presentations of MSF [90]. In addition, hyperbilirubinemia and acute renal failure have been associated with fatal outcome [92]. Concerning regimen-related parameters, administration of fluoroquinolones has been significantly associated with increased disease severity [90]. In 2012, definitions of mild, moderate, severe and malignant forms of the disease were suggested and severity was classified according to clinical and laboratory parameters [93].

Many life-threatening complications of MSF have been described and most of them have the same pathogenetic mechanism, characterized by the angiotropism of *R. conorii* [94]. Cardiac symptoms, including coronary ectasia and atrial fibrillation [95,96], neurological manifestations, such as cerebral infarct, meningoencephalitis and sensorineural hearing loss [97,98,99], renal failure [100], intraocular inflammation [101], pancreatitis [102] and other multi-organ complications have been reported. Children seem to be less susceptible to severe complications of MSF than adults [84]. In two studies in >1000 children in Sicily from 1984–2004, all patients made a complete recovery [78,83]. Finally, other rare complications unrelated to the angiotropism of *R. conorii*, such as secondary hemophagocytic lymphohistiocytosis, might occur during the course of MSF [103].

Clinical suspicion of MSF is of great importance for the practitioners, in order to promptly initiate treatment and avoid the complications of the disease. In this context, a diagnostic score for MSF was developed by Raoult et al. [29]. A restricted part of this score, considering only epidemiological and clinical parameters, was later used to evaluate Tunisian patients and was found to be a useful tool for the presumptive diagnosis of MSF [104].

## 5. Diagnosis

Several laboratory diagnostic tools exist for the diagnosis of *R. conorii* infection that differ in availability, time to obtain results, performance, and the type of information they provide [105]. A thorough knowledge of the advantages and disadvantages of each diagnostic assay is required in order to choose the most appropriate one. Results should always be interpreted in the context of compatible illness in the appropriate epidemiological setting.

Serological tests are widely used for the diagnosis of MSF. The test of choice for the serodiagnosis of the disease is the indirect immunofluorescence antibody (IFA) assay [106]. In this method, the patient’s serum containing the antibodies is added on a slide with fixed rickettsial antigens. The antibodies are then detected by a fluorescein-labeled conjugate. Both IgG and IgM are detectable 7–10 days after symptom onset [107]. In areas where MSF is endemic, IgG titers ≥ 128 and/or IgM titers ≥ 64 are considered indicative of *R. conorii* infection when MSF is suspected [107]. In non-endemic regions IgG titers ≥ 64 and IgM titers ≥ 32 are considered indicative of infection with *Rickettsia* species [107]. At least two serum samples collected two to four weeks apart during acute and convalescent phases of illness are required for definitive diagnosis. Seroconversion or a fourfold or greater rise in antibody titer between acute and convalescent samples offers a confirmation of acute or recent infection [107]. The major drawback of this assay is that sera from patients with MSF usually lack detectable antibodies during the first week of illness and that cross-reactions among the SFG rickettsiae may be observed [106,108]. However, reference laboratories have developed advanced serological techniques to discriminate cross-reacting antibodies in order to identify *Rickettsia* species [107].

The enzyme-linked immunosorbent assay (ELISA) can also be applied for the diagnosis of SFG rickettsioses but can only provide a qualitative measurement, which does not permit the monitoring of antibody titer fluctuations [109]. Western blotting (WB) is useful in detecting antibodies directed against the lipopolysaccharide (LPS) that are produced early in the course of MSF [110]. However, it can yield false positive results due to the fact that the LPS antigen shares common epitopes with other SFG rickettsiae, TG rickettsiae, and certain bacteria [110]. Later in the course of the disease, WB can detect antibodies that are directed against specific rickettsial outer membrane proteins, thus confirming the diagnosis of SFG rickettsioses [107]. Additionally, when it is used in conjunction with cross-absorption, it can aid in the discrimination between the SFG species [105,111].

Culture of rickettsiae represents a challenge owing to the strict intracellular nature of the bacteria. The laboratory isolation of these bacteria requires inoculation onto living cells. The centrifugation shell vial technique (SVT), which was first developed for cytomegalovirus culture, has now been modified for *R. conorii* isolation [112]. The success of this method is dependent on the ratio of microorganisms to cells and the centrifugation step which aids in bacterial adhesion onto the cell lines [113]. Moreover, sampling and inoculation should be performed as soon as possible so as to avoid compromising rickettsial viability [114]. Blood, other sterile body fluids, skin and eschar specimens, and even infected ticks can all be used for culture and should be collected before the initiation of antimicrobial therapy [107,108,114]. Detection of the microorganisms can be carried out using Gimenez or Giemsa staining, immunodetection or PCR [107,108].

Molecular techniques have become increasingly available for the diagnosis of rickettsial diseases. Polymerase chain reaction (PCR) can be used for the early diagnosis of SFG rickettsioses. Both whole blood and tissue specimens, mostly skin specimens, are suitable for PCR amplification [107]. In the absence of severe disease, low numbers of rickettsiae circulate in the blood, and thus the inoculation eschar is a more useful source of rickettsial DNA when it comes to MSF [108,109]. Eschars can be sampled as scrapings, swabs or biopsied [107,115]. The technique for obtaining a swab involves the vigorous swabbing of an unroofed eschar five to six times under sterile conditions [116]. Biopsy specimens should ideally be collected before the onset of treatment since antibiotic therapy might decrease the sensitivity of the assay, possibly due to the decreased number of bacteria in the inoculation site [117]. A variety of different primers have been used for rickettsial DNA identification targeting the *gltA* gene, which encodes the citrate synthase, as well as the genes that encode OmpA and OmpB, and several others [111,118,119]. The *OmpA* gene is specific for the SFG rickettsiae and can therefore be used to exclude a TG organism as a culprit [106,120]. Further characterization of rickettsial species and subspecies is possible using advanced PCR techniques and primers [39], but it is without clinical significance, used only for epidemiological and research purposes.

Finally, another method that can be used for the diagnosis of rickettsioses, before seroconversion, is immunostaining, using immunohistochemistry or immunofluorescence [109]. Samples can be tested after formalin fixation or paraffin embedment. Biopsies can be taken either from the rash or from the eschar lesion. Visualization of the microorganisms is more likely to be successful before or within 48 h of the administration of antibiotic therapy [109]. Immunostaining can also be used in autopsy specimens after fatal cases of MSF, years or decades later [106,107]. It is noteworthy to mention that immunostaining and PCR methods can also be applied on ticks for the detection of rickettsiae [107].

## 6. Treatment

The current treatment for SFG rickettsioses is based on the same antibacterial principles applied over the past decades, as little has changed in this particular field. Regarding the antibiotic susceptibility, it can be assessed through a plaque assay system, but it is not routinely checked and it is only performed in reference laboratories [121]. Thus, the treatment of the disease is mostly based on the administration of empiric antibiotic therapy, even before the diagnostic confirmation, and should be initiated promptly when managing a patient with clinical and epidemiological characteristics suggestive of rickettsiosis [121].

Antibiotics with high intracellular activity such as tetracyclines, chloramphenicol and rifampicin have all been tested in vitro, showing bacteriostatic effect against *R. conorii* [122,123]. Also, some macrolides, such as clarithromycin, azithromycin and josamycin, have demonstrated a similar effect [124]. Josamycin, however, is unavailable in many countries [125]. Finally, fluoroquinolones have a high bactericidal effect when tested in vitro as well [126].

The gold standard treatment for MSF is the administration of doxycycline, the most commonly prescribed antibiotic in such cases [121,127]. Doxycycline is a second-generation tetracycline that has a plethora of advantages, as it is highly effective, cheap, easily accessible, and covers a broad spectrum of bacteria. In addition to that, recent studies have demonstrated that previous doubts regarding the use of doxycycline in certain population groups have been deemed fallacious [128]. Due to its correlation with tetracyclines, doxycycline has been labeled an FDA class D medication, halting its use in pregnant women [129]. Also, in children under eight years of age, there is concern regarding possible permanent tooth discoloration [130]. However, data for adverse effects during doxycycline administration such as teratogenicity, permanent inhibition of bone growth in fetuses and preterm infants, and severe hepatotoxicity are absent, in contrast to previous beliefs [128]. In addition, further studies have demonstrated its safety profile in the young population, as the administration of short courses of doxycycline (up to 21 days) to children under the age of eight neither seems to darken the shade of the teeth nor to cause visible staining [131,132,133].

The hesitancy to use doxycycline, due to the aforementioned alleged adverse effects, could potentially lead to inferior treatments for the management of serious infections [128]. Also, in patients with a history of a non-life-threatening tetracycline-class allergy, the administration of doxycycline in an observed setting should be considered. In case of a previous critical allergic reaction to tetracyclines, options include the rapid doxycycline desensitization (if a history of an immediate hypersensitivity reaction exists) or the use of an alternative antibacterial agent [109].

The most commonly used dosage of doxycycline for the treatment of MSF is 100 mg twice daily for adults, while the pediatric dosage is 2.2 mg/kg every 12 h for children below 45 kg (or adult dosage if >45 kg) [121,127]. Controversial literature exists regarding the appropriate duration of treatment, as studies have demonstrated that even one-day courses of treatment with one or two doses of 200 mg of doxycycline (12 h apart) seem to be as effective as longer courses [121,134]. Nevertheless, the majority of authors recommend that the extent of the treatment should be guided upon the severity of the clinical presentation and the response to treatment [121,127]. We agree with some authors’ recommendation for doxycycline administration of 100 mg twice daily in adults for at least 3 days after fever defervescence, with a minimum course of treatment of 5–7 days [127]. For febrile children, it is also advised that the treatment should be continued for at least three days after fever resolution [131].

Macrolides are efficient and safe alternatives for treating mild to moderate cases of MSF when patients cannot receive doxycycline, as they have the potential to achieve high intracellular concentrations [124,125]. Therefore, clarithromycin, azithromycin and josamycin can all be equally used in children and adults, while azithromycin and josamycin are preferred for pregnant women. Noteworthy, azithromycin is also the preferred regimen for non-compliant patients, as the long half-life of the drug makes feasible a short course of treatment [121,124]. Chloramphenicol has also been used as an alternative to doxycycline for the treatment of MSF [121]. However, the serious toxicity observed with its use and the reported relapses after the course of treatment have limited its use [121,135]. The administration of fluoroquinolones, even though it correlates with favorable in vitro results, is not preferred, as it has been demonstrated to result in inferior outcomes when compared to standard treatment. In particular, it has been reported that fluoroquinolone treatment is associated with increased MSF severity and longer hospital stay [90], while further research demonstrated that the use of ciprofloxacin had a detrimental effect on *R. conorii*-infected host cells [126]. Finally, data derived mainly from Rocky Mountain spotted fever indicate that the use of sulfonamide antibiotics for the treatment of SFG rickettsioses is correlated with unfavorable patient outcomes [109].

A future prospect in the treatment of MSF has been proposed by Chan et al. who studied the molecular basis of rickettsial immunity and demonstrated that the administration of anti-rickettsial monoclonal antibodies could lead to the killing of *R. conorii* in rodents through complement activation [136]. Such an appliance could have a potential future utility, as these monoclonal antibodies could be used as an alternative to antibiotic treatment for rickettsial disease in case of antibiotic resistance or contraindications to antibiotic use.

## 7. Prevention

MSF, as has already been described, is a complex disease. Multiple factors interact to lead to human infection with *R. conorii conorii*. Therefore, prevention strategies should target all of these different pathways that *Rickettsia conorii conorii* go through before finally infecting humans. The epidemic control of MSF is a rather challenging accomplishment, as there are lots of questions regarding the reservoir system of the bacteria, which is still uncharted [137].

Firstly, preventive measures should concern human behavior and emphasize the limitation of exposure to vectors carrying the bacteria. Such measures include the avoidance of tick-infested loci, the regular and thorough check of the human body for tick infestation after the entrance in tick areas, the proper removal of ticks once spotted, and the use of tick repellants. Long-lasting permethrin impregnated clothing for outdoor exposures in areas with high tick populations is quite effective in preventing tick bites [138,139].

Vector control strategies are also useful for the prevention of MSF. In urban and populated areas, preventive measures should focus on tick population control in canines, which can be accomplished via special tick-repellent collars [137]. In rural areas, the use of tick insecticides and acaricides for the treatment of the animals and the environment has been heavily used to set the tick population under control, although the observed resistance to such agents and the negative environmental impact are points of critique [137,140].

There are currently no vaccines commercially available for rickettsioses [141]. Previous attempts for *R. conorii* vaccine development failed to provide long lasting immunity [142]. Also, humans are accidental, and probably dead-end, hosts for SFG rickettsiae, while the majority of MSF cases respond well to antibiotic treatment. Such aspects raise the question of whether the development of vaccination is really essential for this clinical setting [142]. Future prospects for early prevention could be based on the development of anti-tick vaccines that could exploit the complex rickettsiae – tick interactions, having the advantage of being effective against multiple tick-borne diseases. Such a venture requires the detailed comprehension of the molecular cascades and interplays between the bacteria and their vectors. A major challenge regarding the anti-tick vaccine development is the difficulty in predicting the most suitable proteins to target, as the different *Rickettsia* species provoke different host responses [142,143].

Lastly, practitioners should have in mind that antibiotic prophylaxis after a tick bite is not currently recommended for the prevention of rickettsial infections [109]. However, after the prompt and appropriate removal of attached ticks, patients should be encouraged to watch themselves for fever, rash, headache or other symptoms in the subsequent few weeks after the tick bite in order to seek medical advice whenever needed.

## 8. Conclusions

Although our knowledge concerning MSF has evolved over the past few years with the identification of certain subspecies within the *R. conorii* complex, including *R. conorii conorii* as the etiological agent of the disease, it still constitutes an intricate epidemiological entity. A deeper understanding of the reservoir system of these bacteria is needed for the development of targeted preventive measures, since there are currently no vaccines commercially available. The diagnostic assays for MSF have also advanced significantly and allow for an earlier confirmation of the disease. Finally, the recognition of typical and atypical features of MSF by clinicians, both within and outside of the endemic areas, is crucial for the prompt administration of antibiotic treatment.

## References

[1] Rovery C., Brouqui P., Raoult D. (2008). Questions on Mediterranean spotted fever a century after its discovery. Emerg. Infect. Dis..

[2] Paris D.H., Day N.P.J., Farrar J., Hotez P.J., Junghanss T., Kang G., Lalloo D., White N.J. (2014). Tropical rickettsial infections. Manson’s Tropical Diseases.

[3] Delord M., Socolovschi C., Parola P. (2014). Rickettsioses and Q fever in travelers (2004–2013). Travel Med. Infect. Dis..

[4] Jensenius M., Fournier P.E., Raoult D. (2004). Tick-borne rickettsioses in international travellers. Int. J. Infect. Dis..

[5] Parola P., Raoult D. (2001). Ticks and tickborne bacterial diseases in humans: An emerging infectious threat. Clin. Infect. Dis..

[6] Raoult D., Roux V. (1997). Rickettsioses as paradigms of new or emerging infectious diseases. Clin. Microbiol. Rev..

[7] Weinert L.A., Werren J.H., Aebi A., Stone G.N., Jiggins F.M. (2009). Evolution and diversity of *Rickettsia* bacteria. BMC Biol..

[8] Sahni S.K., Narra H.P., Sahni A., Walker D.H. (2013). Recent molecular insights into rickettsial pathogenesis and immunity. Future Microbiol..

[9] Klein D., Beth-Din A., Cohen R., Lazar S., Glinert I., Zayyad H., Atiya-Nasagi Y. (2019). New spotted fever group rickettsia isolate, identified by sequence analysis of conserved genomic regions. Pathogens.

[10] Ogata H., Audic S., Renesto-Audiffren P., Fournier P.E., Barbe V., Samson D., Roux V., Cossart P., Weissenbach J., Claverie J.M. (2001). Mechanisms of evolution in *Rickettsia conorii* and *R. prowazekii*. Science.

[11] Zhu Y., Fournier P.E., Eremeeva M., Raoult D. (2005). Proposal to create subspecies of *Rickettsia conorii* based on multi-locus sequence typing and an emended description of *Rickettsia conorii*. BMC Microbiol..

[12] Karim S., Kumar D., Budachetri K. (2021). Recent advances in understanding tick and rickettsiae interactions. Parasite Immunol..

[13] Walker D.H., Ismail N. (2008). Emerging and re-emerging rickettsioses: Endothelial cell infection and early disease events. Nat. Rev. Microbiol..

[14] Parola P., Socolovschi C., Raoult D. (2009). Deciphering the relationships between *Rickettsia conorii conorii* and *Rhipicephalus sanguineus* in the ecology and epidemiology of Mediterranean spotted fever. Ann. N. Y. Acad. Sci..

[15] Socolovschi C., Gaudart J., Bitam I., Huynh T.P., Raoult D., Parola P. (2012). Why are there so few *Rickettsia conorii conorii*-infected *Rhipicephalus sanguineus* ticks in the wild?. PLoS Negl. Trop. Dis..

[16] Raoult D., Dupont H.T., Chicheportiche C., Peter O., Gilot B., Drancourt M. (1993). Mediterranean spotted fever in Marseille, France: Correlation between prevalence of hospitalized patients, seroepidemiology, and prevalence of infected ticks in three different areas. Am. J. Trop. Med. Hyg..

[17] Fernández-Soto P., Pérez-Sánchez R., Alamo-Sanz R., Encinas-Grandes A. (2006). Spotted fever group rickettsiae in ticks feeding on humans in northwestern Spain: Is *Rickettsia conorii* vanishing?. Ann. N. Y. Acad. Sci..

[18] Marquez F.J., Rodriguez-Liebana J.J., Soriguer R.C., Muniain M.A., Bernabeu-Wittel M., Caruz A., Contreras-Chova F. (2008). Spotted fever group *Rickettsia* in brown dog ticks *Rhipicephalus sanguineus* in southwestern Spain. Parasitol. Res..

[19] Segura-Porta F., Diestre-Ortin G., Ortuno-Romero A., Sanfeliu-Sala I., Font-Creus B., Munoz-Espin T., de Antonio E.M., Casal-Fabrega J. (1998). Prevalence of antibodies to spotted fever group rickettsiae in human beings and dogs from and endemic area of mediterranean spotted fever in Catalonia, Spain. Eur. J. Epidemiol..

[20] Alexandre N., Santos A.S., Bacellar F., Boinas F.J., Núncio M.S., de Sousa R. (2011). Detection of *Rickettsia conorii* strains in Portuguese dogs (*Canis familiaris*). Ticks Tick-Borne Dis..

[21] Kelly P.J., Matthewman L.A., Mason P.R., Courtney S., Katsande C., Rukwava J. (1992). Experimental infection of dogs with a Zimbabwean strain of *Rickettsia conorii*. J. Trop. Med. Hyg..

[22] Le Gac P. (1966). Repercussions of myxomatosis on Mediterranean boutonneuse exanthematic fever. Bull. World Health Organ..

[23] De Sousa R., Nobrega S.D., Bacellar F., Torgal J. (2003). Mediterranean spotted fever in Portugal: Risk factors for fatal outcome in 105 hospitalized patients. Ann. N. Y. Acad. Sci..

[24] Vitale G., Mansuelo S., Rolain J.M., Raoult D. (2006). *Rickettsia massiliae* human isolation. Emerg. Infect. Dis..

[25] Colomba C., Trizzino M., Giammanco A., Bonura C., Di Bona D., Tolomeo M., Cascio A. (2017). Israeli Spotted Fever in Sicily. Description of two cases and minireview. Int. J. Infect. Dis..

[26] Guccione C., Colomba C., Tolomeo M., Trizzino M., Iaria C., Cascio A. (2021). Rickettsiales in Italy. Pathogens.

[27] Pascucci I., Di Domenico M., Curini V., Cocco A., Averaimo D., D’Alterio N., Cammà C. (2019). Diversity of Rickettsia in ticks collected in Abruzzi and Molise regions (central Italy). Microorganisms.

[28] Gilot B., Laforge M.L., Pichot J., Raoult D. (1990). Relationships between the *Rhipicephalus sanguineus* complex ecology and Mediterranean spotted fever epidemiology in France. Eur. J. Epidemiol..

[29] Raoult D., Tissot Dupont H., Caraco P., Brouqui P., Drancourt M., Charrel C. (1992). Mediterranean spotted fever in Marseille: Descriptive epidemiology and the influence of climatic factors. Eur. J. Epidemiol..

[30] Parola P., Paddock C.D., Socolovschi C., Labruna M.B., Mediannikov O., Kernif T., Abdad M.Y., Stenos J., Bitam I., Fournier P.E. (2013). Update on tick-borne rickettsioses around the world: A geographic approach. Clin. Microbiol. Rev..

[31] Parola P., Socolovschi C., Jeanjean L., Bitam I., Fournier P.E., Sotto A., Labauge P., Raoult D. (2008). Warmer weather linked to tick attack and emergence of severe rickettsioses. PLoS Negl. Trop. Dis..

[32] Rovery C., Raoult D. (2008). Mediterranean spotted fever. Infect. Dis. Clin. N. Am..

[33] Macaluso K.R., Sonenshine D.E., Ceraul S.M., Azad A.F. (2002). Rickettsial infection in *Dermacentor variabilis* (Acari: Ixodidae) inhibits transovarial transmission of a second Rickettsia. J. Med. Entomol..

[34] Parker R.R., Spencer R.R. (1926). Rocky mountain spotted fever: A study of the relationship between the presence of Rickettsia-like organisms in tick smears and the infectiveness of the same ticks. Public Health Reports (1896–1970).

[35] Chan Y.G., Riley S.P., Martinez J.J. (2010). Adherence to and invasion of host cells by spotted fever group *Rickettsia* species. Front. Microbiol..

[36] Cardwell M.M., Martinez J.J. (2009). The Sca2 autotransporter protein from *Rickettsia conorii* is sufficient to mediate adherence to and invasion of cultured mammalian cells. Infect. Immun..

[37] Martinez J.J., Seveau S., Veiga E., Matsuyama S., Cossart P. (2005). Ku70, a component of DNA-dependent protein kinase, is a mammalian receptor for *Rickettsia conorii*. Cell.

[38] Blanc G., Ngwamidiba M., Ogata H., Fournier P.-E., Claverie J.-M., Raoult D. (2005). Molecular evolution of *Rickettsia* surface antigens: Evidence of positive selection. Mol. Biol. Evol..

[39] Blanda V., D’Agostino R., Giudice E., Randazzo K., La Russa F., Villari S., Vullo S., Torina A. (2020). New real-time PCRs to differentiate *Rickettsia* spp. and *Rickettsia conorii*. Molecules.

[40] Monferran S., Muller C., Mourey L., Frit P., Salles B. (2004). The membrane-associated form of the DNA repair protein Ku is involved in cell adhesion to fibronectin. J. Mol. Biol..

[41] Lucero H., Gae D., Taccioli G.E. (2003). Novel localization of the DNA-PK complex in lipid rafts: A putative role in the signal transduction pathway of the ionizing radiation response. J. Biol. Chem..

[42] Chan Y.G., Cardwell M.M., Hermanas T.M., Uchiyama T., Martinez J.J. (2009). Rickettsial outer-membrane protein B (rOmpB) mediates bacterial invasion through Ku70 in an actin, c-Cbl, clathrin and caveolin 2-dependent manner. Cell. Microbiol..

[43] Martinez J.J., Cossart P. (2004). Early signaling events involved in the entry of *Rickettsia conorii* into mammalian cells. J. Cell Sci..

[44] Riley S.P., Patterson J.L., Martinez J.J. (2012). The rickettsial OmpB β-peptide of *Rickettsia conorii* is sufficient to facilitate factor H-mediated serum resistance. Infect. Immun..

[45] Hillman R.D., Baktash Y.M., Martinez J.J. (2013). OmpA-mediated rickettsial adherence to and invasion of human endothelial cells is dependent upon interaction with alpha2beta1 integrin. Cell. Microbiol..

[46] Sahni A., Patel J., Narra H.P., Schroeder C.L.C., Walker D.H., Sahni S.K. (2017). Fibroblast growth factor receptor-1 mediates internalization of pathogenic spotted fever rickettsiae into host endothelium. PLoS ONE.

[47] Radulovic S., Troyer J.M., Beier M.S., Lau A.O., Azad A.F. (1999). Identification and molecular analysis of the gene encoding *Rickettsia typhi* hemolysin. Infect. Immun..

[48] Renesto P., Dehoux P., Gouin E., Touqui L., Cossart P., Raoult D. (2003). Identification and characterization of a phospholipase D-superfamily gene in Rickettsiae. J. Infect. Dis..

[49] Whitworth T., Popov V.L., Yu X.J., Walker D.H., Bouyer D.H. (2005). Expression of the *Rickettsia prowazekii* pld or tlyC gene in *Salmonella enterica* serovar typhimurium mediates phagosomal escape. Infect. Immun..

[50] Gouin E., Egile C., Dehoux P., Villiers V., Adams J., Gertler F., Li R., Cossart P. (2004). The RickA protein of *Rickettsia conorii* activates the Arp2/3 complex. Nature.

[51] Heinzen R.A., Grieshaber S.S., Van Kirk L.S., Devin C.J. (1999). Dynamics of actin-based movement by *Rickettsia rickettsii* in vero cells. Infect. Immun..

[52] Van Kirk L.S., Hayes S.F., Heinzen R.A. (2000). Ultrastructure of *Rickettsia rickettsii* actin tails and localization of cytoskeletal proteins. Infect. Immun..

[53] Walker D.H., Gear J.H. (1985). Correlation of the distribution of *Rickettsia conorii*, microscopic lesions, and clinical features in South African tick bite fever. Am. J. Trop. Med. Hyg..

[54] Osterloh A. (2017). Immune response against rickettsiae: Lessons from murine infection models. Med. Microbiol. Immunol..

[55] Rydkina E., Sahni A., Baggs R.B., Silverman D.J., Sahni S.K. (2006). Infection of human endothelial cells with spotted fever group rickettsiae stimulates cyclooxygenase 2 expression and release of vasoactive prostaglandins. Infect. Immun..

[56] Mansueto P., Vitale G., Cascio A., Seidita A., Pepe I., Carroccio A., di Rosa S., Rini G.B., Cillari E., Walker D.H. (2012). New insight into immunity and immunopathology of Rickettsial diseases. Clin. Dev. Immunol..

[57] Sporn L.A., Sahni S.K., Lerner N.B., Marder V.J., Silverman D.J., Turpin L.C., Schwab A.L. (1997). *Rickettsia rickettsii* infection of cultured human endothelial cells induces NF-kappaB activation. Infect. Immun..

[58] Sahni S.K., Van Antwerp D.J., Eremeeva M.E., Silverman D.J., Marder V.J., Sporn L.A. (1998). Proteasome-independent activation of nuclear factor kappaB in cytoplasmic extracts from human endothelial cells by *Rickettsia rickettsii*. Infect. Immun..

[59] Rydkina E., Silverman D.J., Sahni S.K. (2005). Activation of p38 stress-activated protein kinase during *Rickettsia rickettsii* infection of human endothelial cells: Role in the induction of chemokine response. Cell. Microbiol..

[60] Dinarello C.A. (2002). The IL-1 family and inflammatory diseases. Clin. Exp. Rheumatol..

[61] Kaplanski G., Teysseire N., Farnarier C., Kaplanski S., Lissitzky J.C., Durand J.M., Soubeyrand J., Dinarello C.A., Bongrand P. (1995). IL-6 and IL-8 production from cultured human endothelial cells stimulated by infection with *Rickettsia conorii* via a cell-associated IL-1 alpha-dependent pathway. J. Clin. Investig..

[62] Colonne P.M., Eremeeva M.E., Sahni S.K. (2011). Beta interferon-mediated activation of signal transducer and activator of transcription protein 1 interferes with *Rickettsia conorii* replication in human endothelial cells. Infect. Immun..

[63] Zhao Y., Valbuena G., Walker D.H., Gazi M., Hidalgo M., DeSousa R., Oteo J.A., Goez Y., Brasier A.R. (2016). Endothelial cell proteomic response to *Rickettsia conorii* infection reveals activation of the Janus Kinase (JAK)-Signal Transducer and Activator of Transcription (STAT)-Inferferon Stimulated Gene (ISG)15 pathway and reprogramming plasma membrane integrin/cadherin signaling. Mol. Cell. Proteom. MCP.

[64] Feng H.M., Walker D.H. (2000). Mechanisms of intracellular killing of *Rickettsia conorii* in infected human endothelial cells, hepatocytes, and macrophages. Infect. Immun..

[65] Feng H.M., Popov V.L., Walker D.H. (1994). Depletion of gamma interferon and tumor necrosis factor alpha in mice with *Rickettsia conorii*-infected endothelium: Impairment of rickettsicidal nitric oxide production resulting in fatal, overwhelming rickettsial disease. Infect. Immun..

[66] Narra H.P., Sahni A., Khanipov K., Fofanov Y., Sahni S.K. (2019). Global transcriptomic profiling of pulmonary gene expression in an experimental murine model of *Rickettsia conorii* infection. Genes.

[67] Jordan J.M., Woods M.E., Feng H.M., Soong L., Walker D.H. (2007). Rickettsiae-stimulated dendritic cells mediate protection against lethal rickettsial challenge in an animal model of spotted fever rickettsiosis. J. Infect. Dis..

[68] Curto P., Santa C., Allen P., Manadas B., Simões I., Martinez J.J. (2019). A pathogen and a non-pathogen spotted fever group rickettsia trigger differential proteome signatures in macrophages. Front. Cell. Infect. Microbiol..

[69] Allen P.E., Noland R.C., Martinez J.J. (2021). *Rickettsia conorii* survival in THP-1 macrophages involves host lipid droplet alterations and active rickettsial protein production. Cell. Microbiol..

[70] Curto P., Riley S.P., Simões I., Martinez J.J. (2019). Macrophages infected by a pathogen and a non-pathogen spotted fever group *Rickettsia* reveal differential reprogramming signatures early in infection. Front. Cell. Infect. Microbiol..

[71] Sahni A., Narra H.P., Sahni S.K. (2020). Activation of mechanistic target of rapamycin (mTOR) in human endothelial cells infected with pathogenic spotted fever group rickettsiae. Int. J. Mol. Sci..

[72] Chowdhury I.H., Narra H.P., Sahni A., Khanipov K., Fofanov Y., Sahni S.K. (2019). Enhancer associated long non-coding RNA transcription and gene regulation in experimental models of rickettsial infection. Front. Immunol..

[73] Patel J.G., Narra H.P., Sepuru K.M., Sahni A., Golla S.R., Sahni A., Singh A., Schroeder C.L.C., Chowdhury I.H., Popov V.L. (2020). Evolution, purification, and characterization of RC0497: A peptidoglycan amidase from the prototypical spotted fever species *Rickettsia conorii*. Biol. Chem..

[74] Zhao Y., Fang R., Zhang J., Zhang Y., Bechelli J., Smalley C., Valbuena G., Walker D.H., Oteo J.A., Brasier A.R. (2020). Quantitative proteomics of the endothelial secretome identifies RC0497 as diagnostic of acute rickettsial spotted fever infections. Am. J. Pathol..

[75] Martín Farfán A., Juárez Fernández C., Calbo Torrecillas F., Porras Ballesteros J., Díaz Recio M., Bermúndez Recio F. (1985). Clinico-epidemiological study of 164 cases of boutonneuse fever. Rev. Clin. Esp..

[76] Crespo P., Seixas D., Marques N., Oliveira J., da Cunha S., Melico-Silvestre A. (2015). Mediterranean spotted fever: Case series of 24 years (1989–2012). SpringerPlus.

[77] Anton E., Font B., Munoz T., Sanfeliu I., Segura F. (2003). Clinical and laboratory characteristics of 144 patients with Mediterranean spotted fever. Eur. J. Clin. Microbiol. Infect. Dis..

[78] Colomba C., Saporito L., Polara V.F., Rubino R., Titone L. (2006). Mediterranean spotted fever: Clinical and laboratory characteristics of 415 Sicilian children. BMC Infect. Dis..

[79] Raoult D., Weiller P.J., Chagnon A., Chaudet H., Gallais H., Casanova P. (1986). Mediterranean spotted fever: Clinical, laboratory and epidemiological features of 199 cases. Am. J. Trop. Med. Hyg..

[80] López Parés P., Muñoz Espín T., Espejo Arenas E., Font Creus B., Segura Porta F., Martínez Vila I., Travería Casanova J., Bella Cueto F. (1988). Mediterranean spotted fever in childhood. Prospective study of 130 cases. An. Esp. Pediatr..

[81] Vitaliti G., Falsaperla R., Lubrano R., Rapisarda V., Cocuzza S., Nunnari G., Pavone P. (2015). Incidence of Mediterranean spotted fever in Sicilian children: A clinical-epidemiological observational retrospective study from 1987 to 2010. Int. J. Infect. Dis..

[82] Peixoto S., Ferreira J., Carvalho J., Martins V. (2018). Mediterranean spotted fever in children: Study of a Portuguese endemic region. Acta Med. Port..

[83] Cascio A., Dones P., Romano A., Titone L. (1998). Clinical and laboratory findings of boutonneuse fever in Sicilian children. Eur. J. Pediatr..

[84] Baltadzhiev I., Kevorkyan A., Popivanova N. (2020). Mediterranean spotted fever in child and adult patients: Investigation from an endemic region in Bulgaria. Cent. Eur. J. Public Health.

[85] Herrador Z., Fernandez-Martinez A., Gomez-Barroso D., Leon I., Vieira C., Muro A., Benito A. (2017). Mediterranean spotted fever in Spain, 1997–2014: Epidemiological situation based on hospitalization records. PLoS ONE.

[86] Mansueto S., Vitale G., Miceli M.D., Tringali G., Quartararo P., Picone D.M., Occhino C. (1984). A sero-epidemiological survey of asymptomatic cases of Boutonneuse fever in western Sicily. Trans. R. Soc. Trop. Med. Hyg..

[87] Raoult D., Nicolas D., De Micco P., Gallais H., Casanova P. (1985). Epidemiologic aspects of Mediterranean Boutonneuse fever in the south of Corsica. Bull. Soc. Pathol. Exot. Fil..

[88] Demeester R., Claus M., Hildebrand M., Vlieghe E., Bottieau E. (2010). Diversity of life-threatening complications due to Mediterranean spotted fever in returning travelers. J. Travel Med..

[89] Raoult D., Zuchelli P., Weiller P.J., Charrel C., San Marco J.L., Gallais H., Casanova P. (1986). Incidence, clinical observations and risk factors in the severe form of Mediterranean spotted fever among patients admitted to hospital in Marseilles 1983–1984. J. Infect..

[90] Botelho-Nevers E., Rovery C., Richet H., Raoult D. (2011). Analysis of risk factors for malignant Mediterranean spotted fever indicates that fluoroquinolone treatment has a deleterious effect. J. Antimicrob. Chemother..

[91] Piras M.A., Calia G., Saba F., Gakis C., Andreoni G. (1983). Glucose-6-phosphate dehydrogenase deficiency in male patients with Mediterranean spotted fever in Sardinia. J. Infect. Dis..

[92] Sousa R., Franca A., Doria Nobrega S., Belo A., Amaro M., Abreu T., Pocas J., Proenca P., Vaz J., Torgal J. (2008). Host- and microbe-related risk factors for and pathophysiology of fatal *Rickettsia conorii* infection in Portuguese patients. J. Infect. Dis..

[93] Baltadzhiev I.G., Popivanova N.I., Stoilova Y.M., Kevorkian A.K. (2012). Mediterranean spotted fever–classification by disease course and criteria for determining the disease severity. Folia Med. (Plovdiv).

[94] Walker D.H., Herrero-Herrero J.I., Ruiz-Beltran R., Bullon-Sopelana A., Ramos-Hidalgo A. (1987). The pathology of fatal Mediterranean spotted fever. Am. J. Clin. Pathol..

[95] Cascio A., Maggio M.C., Cardella F., Zangara V., Accomando S., Costa A., Iaria C., Mansueto P., Giordano S. (2011). Coronary involvement in Mediterranean spotted fever. New Microbiol..

[96] Colomba C., Saporito L., Colletti P., Mazzola G., Rubino R., Pampinella D., Titone L. (2008). Atrial fibrillation in Mediterranean spotted fever. J. Med. Microbiol..

[97] Botelho-Nevers E., Foucault C., Lepidi H., Brouqui P. (2005). Cerebral infarction: An unusual complication of Mediterranean spotted fever. Eur. J. Intern. Med..

[98] Bougteba A., Basir A., Charradi N. (2011). Meningoencephalitis caused by *Rickettsia conorii* in a young infant. Rev. Neurol..

[99] Tsiachris D., Deutsch M., Vassilopoulos D., Zafiropoulou R., Archimandritis A.J. (2008). Sensorineural hearing loss complicating severe rickettsial diseases: Report of two cases. J. Infect..

[100] Montasser D.I., Zajjari Y., Alayoud A., Bahadi A., Aatif T., Hassani K., Hamzi A., Allam M., Benyahia M., Oualim Z. (2011). Acute renal failure as a complication of Mediterranean spotted fever. Nephrol. Ther..

[101] Agahan A.L., Torres J., Fuentes-Paez G., Martinez-Osorio H., Orduna A., Calonge M. (2011). Intraocular inflammation as the main manifestation of *Rickettsia conorii* infection. Clin. Ophthalmol..

[102] Rombola F. (2011). Mediterranean spotted fever presenting as an acute pancreatitis. Acta Gastroenterol. Belg..

[103] Cascio A., Giordano S., Dones P., Venezia S., Iaria C., Ziino O. (2011). Haemophagocytic syndrome and rickettsial diseases. J. Med. Microbiol..

[104] Letaief A., Souissi J., Trabelsi H., Ghannem H., Jemni L. (2003). Evaluation of clinical diagnosis scores for Boutonneuse fever. Ann. N. Y. Acad. Sci..

[105] Fang R., Blanton L.S., Walker D.H. (2017). Rickettsiae as emerging infectious agents. Clin. Lab. Med..

[106] La Scola B., Raoult D. (1997). Laboratory diagnosis of rickettsioses: Current approaches to diagnosis of old and new rickettsial diseases. J. Clin. Microbiol..

[107] Portillo A., de Sousa R., Santibanez S., Duarte A., Edouard S., Fonseca I.P., Marques C., Novakova M., Palomar A.M., Santos M. (2017). Guidelines for the detection of *Rickettsia* spp.. Vector-Borne Zoonotic Dis..

[108] Brouqui P., Bacellar F., Baranton G., Birtles R.J., Bjoersdorff A., Blanco J.R., Caruso G., Cinco M., Fournier P.E., Francavilla E. (2004). Guidelines for the diagnosis of tick-borne bacterial diseases in Europe. Clin. Microbiol. Infect..

[109] Biggs H.M., Behravesh C.B., Bradley K.K., Dahlgren F.S., Drexler N.A., Dumler J.S., Folk S.M., Kato C.Y., Lash R.R., Levin M.L. (2016). Diagnosis and management of tickborne rickettsial diseases: Rocky mountain spotted fever and other spotted fever group rickettsioses, ehrlichioses, and anaplasmosis—United States. MMWR Recomm. Rep..

[110] Teysseire N., Raoult D. (1992). Comparison of Western immunoblotting and microimmunofluorescence for diagnosis of Mediterranean spotted fever. J. Clin. Microbiol..

[111] Robinson M.T., Satjanadumrong J., Hughes T., Stenos J., Blacksell S.D. (2019). Diagnosis of spotted fever group *Rickettsia* infections: The Asian perspective. Epidemiol. Infect..

[112] Marrero M., Raoult D. (1989). Centrifugation-shell vial technique for rapid detection of Mediterranean spotted fever rickettsia in blood culture. Am. J. Trop. Med. Hyg..

[113] Gouriet F., Fenollar F., Patrice J.Y., Drancourt M., Raoult D. (2005). Use of shell-vial cell culture assay for isolation of bacteria from clinical specimens: 13 years of experience. J. Clin. Microbiol..

[114] La Scola B., Raoult D. (1996). Diagnosis of Mediterranean spotted fever by cultivation of *Rickettsia conorii* from blood and skin samples using the centrifugation-shell vial technique and by detection of *R. conorii* in circulating endothelial cells: A 6-year follow-up. J. Clin. Microbiol..

[115] Bechah Y., Socolovschi C., Raoult D. (2011). Identification of rickettsial infections by using cutaneous swab specimens and PCR. Emerg. Infect. Dis..

[116] Mouffok N., Socolovschi C., Renvoise A., Parola P., Raoult D. (2011). Diagnosis of rickettsioses from eschar swab samples, Algeria. Emerg. Infect. Dis..

[117] Angelakis E., Richet H., Rolain J.M., La Scola B., Raoult D. (2012). Comparison of real-time quantitative PCR and culture for the diagnosis of emerging Rickettsioses. PLoS Negl. Trop. Dis..

[118] Roux V., Raoult D. (2000). Phylogenetic analysis of members of the genus *Rickettsia* using the gene encoding the outer-membrane protein rOmpB (ompB). Int. J. Syst. Evol. Microbiol..

[119] Ishikura M., Ando S., Shinagawa Y., Matsuura K., Hasegawa S., Nakayama T., Fujita H., Watanabe M. (2003). Phylogenetic analysis of spotted fever group rickettsiae based on gltA, 17-kDa, and rOmpA genes amplified by nested PCR from ticks in Japan. Microbiol. Immunol..

[120] Roux V., Fournier P.E., Raoult D. (1996). Differentiation of spotted fever group rickettsiae by sequencing and analysis of restriction fragment length polymorphism of PCR-amplified DNA of the gene encoding the protein rOmpA. J. Clin. Microbiol..

[121] Botelho-Nevers E., Socolovschi C., Raoult D., Parola P. (2012). Treatment of *Rickettsia* spp. infections: A review. Expert Rev. Anti-Infect. Ther..

[122] Raoult D., Roussellier P., Vestris G., Tamalet J. (1987). In vitro antibiotic susceptibility of *Rickettsia rickettsii* and *Rickettsia conorii*: Plaque assay and microplaque colorimetric assay. J. Infect. Dis..

[123] Rolain J.M., Maurin M., Vestris G., Raoult D. (1998). In vitro susceptibilities of 27 rickettsiae to 13 antimicrobials. Antimicrob. Agents Chemother..

[124] Cascio A., Colomba C., Antinori S., Paterson D.L., Titone L. (2002). Clarithromycin versus azithromycin in the treatment of Mediterranean spotted fever in children: A randomized controlled trial. Clin. Infect. Dis..

[125] Anton E., Munoz T., Traveria F.J., Navarro G., Font B., Sanfeliu I., Segura F. (2015). Randomized trial of clarithromycin for Mediterranean spotted fever. Antimicrob. Agents Chemother..

[126] Botelho-Nevers E., Edouard S., Leroy Q., Raoult D. (2012). Deleterious effect of ciprofloxacin on *Rickettsia conorii*-infected cells is linked to toxin-antitoxin module up-regulation. J. Antimicrob. Chemother..

[127] Blanton L.S. (2019). The Rickettsioses: A practical update. Infect. Dis. Clin. N. Am..

[128] Cross R., Ling C., Day N.P., McGready R., Paris D.H. (2016). Revisiting doxycycline in pregnancy and early childhood—Time to rebuild its reputation?. Expert Opin. Drug Saf..

[129] Nahum G.G., Uhl K., Kennedy D.L. (2006). Antibiotic use in pregnancy and lactation: What is and is not known about teratogenic and toxic risks. Obstet. Gynecol..

[130] Doryx Drug Label. https://www.accessdata.fda.gov/drugsatfda_docs/label/2015/050582s029lbl.pdf.

[131] Todd S.R., Dahlgren F.S., Traeger M.S., Beltran-Aguilar E.D., Marianos D.W., Hamilton C., McQuiston J.H., Regan J.J. (2015). No visible dental staining in children treated with doxycycline for suspected Rocky Mountain Spotted Fever. J. Pediatr..

[132] Pöyhönen H., Nurmi M., Peltola V., Alaluusua S., Ruuskanen O., Lähdesmäki T. (2017). Dental staining after doxycycline use in children. J. Antimicrob. Chemother..

[133] Stultz J.S., Eiland L.S. (2019). Doxycycline and tooth discoloration in children: Changing of recommendations based on evidence of safety. Ann. Pharmacother..

[134] Bella-Cueto F., Font-Creus B., Segura-Porta F., Espejo-Arenas E., Lopez-Pares P., Munoz-Espin T. (1987). Comparative, randomized trial of one-day doxycycline versus 10-day tetracycline therapy for Mediterranean spotted fever. J. Infect. Dis..

[135] Shaked Y., Samra Y., Maier M.K., Rubinstein E. (1989). Relapse of rickettsial Mediterranean spotted fever and murine typhus after treatment with chloramphenicol. J. Infect..

[136] Chan Y.G., Riley S.P., Chen E., Martinez J.J. (2011). Molecular basis of immunity to rickettsial infection conferred through outer membrane protein B. Infect. Immun..

[137] Kazar J., Brezina R. (1991). Control of rickettsial diseases. Eur. J. Epidemiol..

[138] Faulde M.K., Rutenfranz M., Keth A., Hepke J., Rogge M., Gorner A. (2015). Pilot study assessing the effectiveness of factory-treated, long-lasting permethrin-impregnated clothing for the prevention of tick bites during occupational tick exposure in highly infested military training areas, Germany. Parasitol. Res..

[139] Vaughn M.F., Funkhouser S.W., Lin F.C., Fine J., Juliano J.J., Apperson C.S., Meshnick S.R. (2014). Long-lasting permethrin impregnated uniforms: A randomized-controlled trial for tick bite prevention. Am. J. Prev. Med..

[140] Walker A.R. (2011). Eradication and control of livestock ticks: Biological, economic and social perspectives. Parasitology.

[141] Osterloh A. (2020). The neglected challenge: Vaccination against rickettsiae. PLoS Negl. Trop. Dis..

[142] Rego R.O.M., Trentelman J.J.A., Anguita J., Nijhof A.M., Sprong H., Klempa B., Hajdusek O., Tomas-Cortazar J., Azagi T., Strnad M. (2019). Counterattacking the tick bite: Towards a rational design of anti-tick vaccines targeting pathogen transmission. Parasites Vectors.

[143] Petchampai N., Sunyakumthorn P., Banajee K.H., Verhoeve V.I., Kearney M.T., Macaluso K.R. (2015). Identification of host proteins involved in rickettsial invasion of tick cells. Infect. Immun..

